# Epidemiology of Pediatric Bite/Sting Injuries. One-Year Study of a Pediatric Emergency Department in Israel

**DOI:** 10.1100/tsw.2006.131

**Published:** 2006-06-14

**Authors:** Michal Hemmo-Lotem, Yoav Barnea, Claudia Jinich-Aronowitz, Liri Endy-Findling, David Leshem, Arik Zaretski, Joav Merrick

**Affiliations:** ^1^ Beterem, National Center for Child Safety and Health, Box 7050, IL-49170 Petach Tiqva, Israel; ^2^ School of Public Health, Haifa University, Haifa, Israel; ^3^ Department of Plastic and Reconstructive Surgery, Tel Aviv Sourasky Medical Center, Sackler School of Medicine, Tel Aviv University, Tel Aviv, Israel; ^4^ National Institute of Child Health and Human Development, Beer-Sheva, Israel; ^5^ Center for Multidisciplinary Research in Aging, Faculty of Health Sciences, Ben Gurion University, Beer-Sheva, Israel; ^6^ Office of the Medical Director, Division for Mental Retardation, Ministry of Social Affairs, Jerusalem, Israel

**Keywords:** animal bites, sting injuries, bite injuries, pediatric injuries, dog bites, prevention, Israel

## Abstract

Animal bite/sting injuries are a known source of morbidity with a significantly higher incidence among children who are most often bitten in the face, head, and neck. The objective of this study was to provide a better understanding of bite/sting injuries treated at the pediatric emergency department in order to guide preventive efforts.The sociodemographic, epidemiological, and clinical data on all bite/sting injuries treated in one representative pediatric emergency department in Israel over a 1-year period were retrieved and analyzed. Two hundred of the 9,309 pediatric trauma cases treated in the emergency department were bite/sting injuries (2.1%). Non-Jewish patients were under-represented in this subgroup. The majority of patients were males (61.5%). Age distribution from 0–12 years was fairly even, except for an unexplained peak at 8 years. Dogs inflicted 56%, cats 11%, and hornets 9.5% of the injuries. Limbs were affected in 64% and the head and neck in 27%. Specialists, mostly plastic surgeons, were consulted in 42 cases (21%). The incidence rate for hospitalization (7%) was similar to that seen in other types of injuries. Children with scorpion or hornet stings and young age were more likely to be hospitalized. Preventive and educational aspects are discussed.

## INTRODUCTION

Bite/sting injuries are known sources of morbidity resulting in pain, anxiety, scarring, disfigurement, and wound infections, as well as the emotional impact they invariably have on the victims. In severe forms, they can even result in fatal outcomes[1]. The reported incidence of bite/sting injuries treated at pediatric emergency departments (ED) in Israel is about 4% of all trauma injuries in children[2]. The primary species implicated in animal injuries worldwide are dogs[3,4,5,6,7,8]. There is an estimated 4.5 million dog bites annually in the U.S. to people of all ages with 750,000 of them serious enough to require medical treatment and a 4% hospitalization rate[7,8,9,10]. The incidence of animal injuries is significantly higher among children, especially younger children who are most often bitten in the face, head, and neck[3,4,9,10,11,12,13]. Their injuries range from superficial abrasion and scratches to deep lacerations, resulting in disfiguring tissue losses and even death in an estimated 0.0025% of the cases[1].

The present study is an epidemiological study conducted in Israel in order to determine the incidence and describe the characteristics of bite/sting injuries in children in a representative pediatric ED. The purpose was to provide understanding for preventive efforts.

## METHODS

The study took place at the Schneider Children's Medical Center of Israel, Petach Tiqva, in central Israel with a collection area of approximately 700,000 individuals. The hospital is the largest tertiary pediatric care facility in the country, representative of the national picture insofar as it includes an urban community, a number of Arab villages, and Israeli rural collectives (kibbutzim and moshavim), and is 10 km from the Mediterranean Sea, thus providing easy access to waterborne sources of bites and stings. It accounts for 9% of the pediatric ED admissions and 17% of pediatric hospitalizations nationwide.

We retrospectively reviewed all the childhood injury admissions and retrieved the bite/sting injuries referred to Schneider's pediatric ED throughout a 1-year period. Data on the nature and extent of the injuries, including their source and location, were reviewed and analyzed. Information on the victims included the sociodemographic characteristics of age, sex, and ethnicity. Clinical characteristics included the diagnostic tests performed, the treatment administered, specialist consultations, and recommendations to discharge or admit to hospital. These data were compared to the comprehensive picture of trauma visits to the pediatric ED.

### Statistical Analysis

All data analyses were carried out by SPSS PC version 9.1. Procedures included cross-tabulation, frequency analyses, analysis of variance, and multiple logistic regression with $p < 0.05$ considered significant.

## RESULTS

Of a total 9,309 childhood injury visits to the pediatric ED throughout the 1-year study period, 200 involved bite/sting injuries (2.1%). Most of the patients were referred to the ED from a first-aid station or community clinics. Bite/sting injuries were the eighth cause of trauma ED referral after general cuts and bruises, falls, traffic accidents, sport injuries, object penetration, burns, and poisoning.

The majority of the patients were males (61.5%), but this over-representation of males was similar to that of other injuries (62.9%). The 4% of the patients who were non-Jews was an under-representation of this subgroup compared to the 8.5% figure for all other injuries ($p < 0.03$) (see Table 1).

The age of the victims ranged fairly evenly from 0–12 years and then tapered rapidly towards 17 years of age (mean age, 7.9 ± 4.2 years) with an unexplained peak at age 8 years (12%). Bite/sting injury victims were significantly older than the other trauma patients seen in the ED ($p < 0.001$). They were also more likely to come directly to the ED as opposed to other pediatric trauma patients, usually referred to the ED after examination by someone else, i.e., family physician, pediatrician, school nurse ($p < 0.01$) (see Table 1).

**TABLE 1**
**Comparing Animal Injuries with Other Injuries**

| | Animal Injuries | Other Injuries | *p** |
|---|---|---|---|
| Age (years) | Mean 7.8 (SD 4.2) | Mean 6.4 (SD 4.5) | 0.00** |
| Sex ratio (male:female) | 1.7 | 1.6 | NS |
| % Non-Jews | 4.0% | 8.5% | 0.03 |
| % Examined before presenting to the ED | 63.2% | 73.9% | 0.01 |
| % Hospitalized | 7.1% | 7.0% | NS |
| % Vaccinated (tetanus) | 31.5 | 4.4 | 0.00 |
| % Seen by a plastic surgeon | 6.0 | 3.8 | NS |

*Chi-square test ($p < 0.05$); **students t-test ($p < 0.05$); NS – not significant.

Over one-half of the attacking animals were dogs (56%). Cat bites and scratches accounted for 11% of the cases, and hornets and scorpion stings accounted for 9.5% and 7.5%, respectively (see Fig. 1). There was a significant gender difference between the patients in terms of the culpable species ($p < 0.04$): females were more likely to be bitten or scratched by cats ($p < 0.01$) and males more likely stung by hornets ($p < 0.03$) (see Fig. 1). Non-Jewish children were more likely to be stung by scorpions than Jewish children ($p < 0.001$).

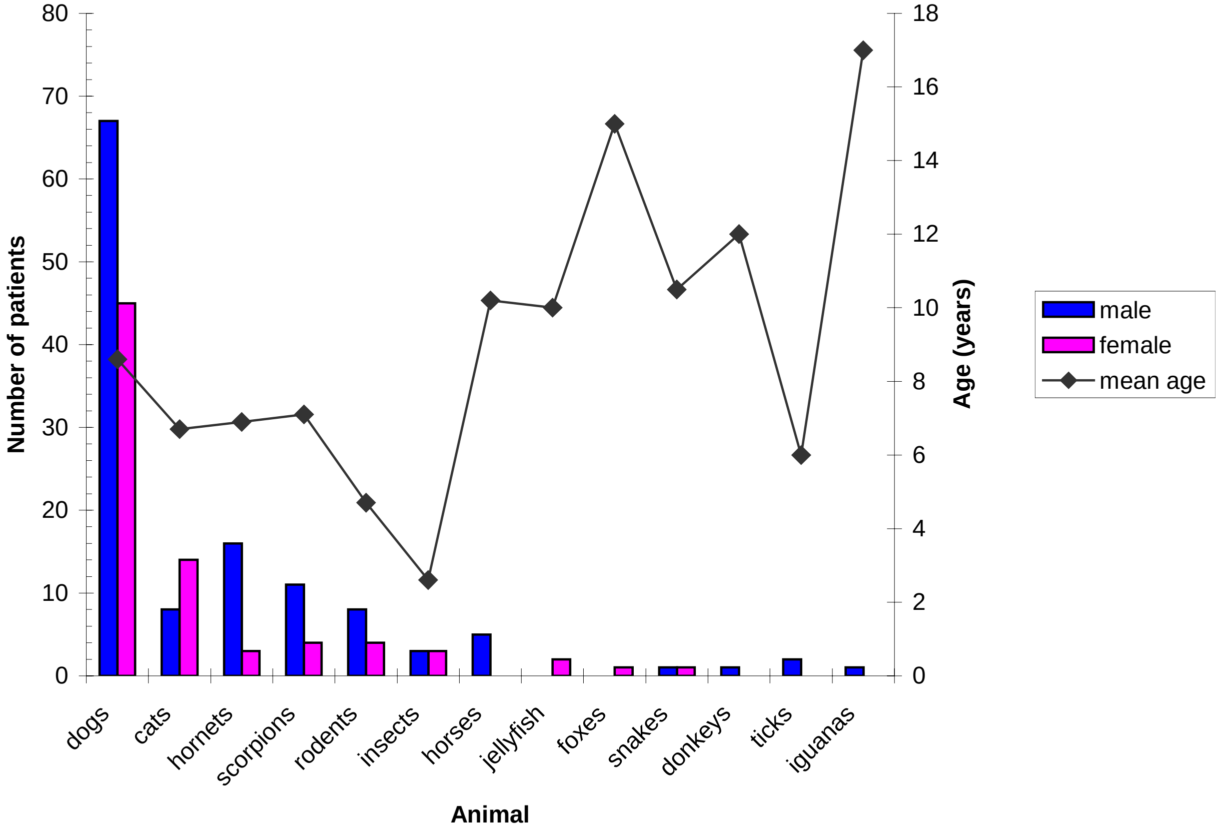

**FIGURE 1**. Distribution of sources of pediatric bite/sting injuries.

There was a significant difference between the mean age of the patients and the animal species ($p < 0.001$). Younger children were attacked by insects and rodents (mean ages 2.6 and 4.6 years, respectively), while older children were attacked by dogs, scorpions, hornets, cats, and ticks (mean ages 8.6, 7.1, 6.9, 6.7, and 6.0 years, respectively) (see Fig. 1).

Most of the patients suffered injuries to the limbs (63%), 28% were injured in the head and neck, and the torso was affected in the remaining 9%. Six patients (3%) presented with injuries to more than one area. There was a significant association between the part of body that was injured (i.e., head, torso, and limbs) and the species of the attacking animal ($p < 0.001$). The substantial injuries to the head and neck were sustained by the victims of dogs, cats, hornets, insects, and donkeys, while victims of scorpions and horses suffered from injuries to the torso. Neither the sex nor age of the patients was related to the injured body part.

Diagnostic tests were performed on 28 patients (14%) and included X-rays (39%), blood tests (36%), ECG, and urine analysis (21% each). The diagnostic test performed was related to the type of animal involved ($p < 0.001$): dog bite victims required more X-rays, while patients who had suffered scorpion stings underwent an ECG.

Overall, 151 patients (76%) received medical treatment in the ED, and 26% received more than one type of treatment. Treatment included antitetanus vaccine (50%), disinfecting of the injured area (37%), bandaging and suturing of open wounds (21%), and systemic medication (10%). Seven patients were detained in the ED in order to undergo medical supervision. One dog bite victim required surgery and another needed a blood transfusion. There was a significant association between the treatment in the ED and the attacking animal ($p < 0.001$). Dog bite victims were the only patients who required suturing of the open wound. They also required significantly more sterilizing procedures ($p < 0.01$) and rabies vaccination than other animal bite victims ($p < 0.001$). Victims of hornet stings received more systemic medications than the others ($p < 0.001$), while scorpion sting victims were more likely to remain in the ED for medical supervision ($p < 0.001$).

Specialist consultants were summoned to the pediatric ED for 42 patients (21%), with two patients needing more than one consultant. In 31%, the specialists were plastic surgeons and in 17%, they were ophthalmologists and orthopedic or general surgeons. There was an overall significant difference according to the species of the attacking animal ($p < 0.001$): dog bite victims required more plastic surgery consultants than other cases ($p < 0.001$) and scorpion sting victims required toxin specialists ($p < 0.001$). Only victims of hornet stings and cat scratches required an ophthalmologist.

Recommendations on discharge were made in 165 cases (82.5%). Eighty patients received more than one recommendation. Antirabies vaccination was considered as being indicated and referrals to the Ministry of Health for further follow-up and decision to vaccinate were made in 52.1% of the cases. Most patients (65%) were referred to their attending physician for medical follow-up. Systemic medication on discharge was recommended to 101 patients, three-fourths of which were oral antibiotic regimens.

Fourteen patients (7%) were hospitalized due to bite/sting injuries, a figure identical to the admittance rate due to other injuries. Ten patients (71% of those hospitalized) were admitted to the pediatric ward, three (21%) to the pediatric surgery department, and one (7%) to the plastic surgery department. Furthermore, plastic surgeons were summoned for medical follow-up to four of the hospitalized patients in the pediatric ward and the pediatric surgery ward.

The hospital admissions rate was not significantly different (~7.0%) compared to that for other types of injuries. The small number of patients hospitalized (n = 14) precluded the use of the hospitalization rate as an indicator of the severity of the injury. A multiple logistic regression model was used in an attempt to predict hospitalization using animal species, age, sex, and ethnicity as predicting variables. Whereas ethnicity and sex did not seem to predict hospitalization, animal species (Odds Ratio = 3.5) and age ($p < 0.02$) did. Patients stung by scorpions or hornets were hospitalized more often than others, and hospitalized children were significantly younger than the children deemed not to require hospitalization (mean ages of 5.3 and 8.1 years, respectively).

In an attempt to categorize the most likely victims of animal attacks, a logistic regression was performed using age, sex, and ethnicity as predicting variables and bite/sting attack victims vs. all other injuries as the dependent variable. While the overall model was significant ($p < 0.001$), only age and ethnicity predicted the likelihood of animal attacks at a level of 0.05. Toddlers were more likely to be attacked by insects ($p < 0.001$) and Jews were twice as likely as non-Jews to suffer bites/stings as opposed to other types of trauma (Odds Ratio = 2.0). Gender did not predict any likelihood of bite/sting injuries.

## DISCUSSION

This is one of the first epidemiological studies of bite/sting injuries treated in a pediatric emergency facility in Israel. Israeli children are exposed to diverse sources of bites and stings. Household pets in Jewish homes are as popular as they are in western countries, the once almost entirely desert terrain is home to scorpions and iguanas, the Mediterranean coast is seasonally infested by jellyfish, and the potentially hazardous insects (such as hornets, bees, and ticks) are commonplace as well. Our intent was to provide data necessary to tailor preventative measures.

### General Characteristics

Gender was a significant factor in bite/sting-type injuries: females were more likely to be scratched or bitten by cats, while males were more likely to be stung by hornets. The finding of non-Jews being significantly under-represented could be due to the low percent of household pet (see below) owners in the non-Jewish community compared to the Jewish one. The average age of the study children was significantly older compared to children who presented with other trauma. We speculate that this may be due to the fact that older children are more confident when approaching and interacting with animals, less intensely supervised by their parents, and not always aware of the dangers that may accompany such an interaction. Age was directly linked to the attacking animal species: older children were more likely to be injured by dogs and cats, and toddlers were more likely to be injured by rodents and insects.

Dogs were the most prevalent attacking animals: they were responsible for the injuries in over one-half of the study population (56%). Non-Jews were more likely to be referred to the ED due to scorpion stings, probably due to their greater representation in rural areas.

### Findings, Tests, Treatment, and the Emergency Department

Most of the attacks were to the limbs. Victims of dogs, cats, hornets, and insects suffered more substantial injuries to the head and neck, while victims of scorpions and horses suffered from injuries to the torso.

The diagnostic tests performed and treatment given in the ED were mostly influenced by the attacking animal species, which also determined the specialist consultation in the ED and the recommendations given on discharge. Although we had documented treatment for only 76% (151) of the study patients, it is reasonable to assume that most patients referred to the ED received some kind of treatment. More than one-half (52%) of the discharge recommendations included a referral to the local health bureau of the Ministry of Health for thorough investigation of the case and, if necessary, for the administration of antirabies vaccination. The absolute majority of the patients referred to the local health bureau of the Ministry of Health were due to dog bite injuries.

Most of the injuries were caused by dogs to the children's limbs and did not require hospitalization. Treatment consisted mostly of antitetanus shots and copiously irrigating and disinfecting the wound. Most of the patients were released from the ED with recommendation to receive a regimen of antibiotics.

## Hospitalization

The hospitalization rate was 7%, similar to the overall admittance rate in other childhood injuries, but higher than reported in the literature (up to 5%)[10,13]. Most of the hospitalized patients (71%) were admitted to the pediatric ward mainly for systemic treatment and observation following scorpion or hornet bites (these patients were 3.5 times more likely to be hospitalized for close medical supervision). The rest of the patients were admitted to the pediatric and pediatric surgery department for wound care and observation. One patient was admitted to the plastic surgery department and four other patients were medically followed-up by plastic surgeons in the pediatric and pediatric surgery department. Younger children were also more likely to be hospitalized, probably for supervision due to their more fragile state.

## Study Limitations

The bite/sting injuries seen at the pediatric emergency facilities comprise essentially all the types of attacks in the country. It would, however, be reasonable to expect a far greater incidence of attacks by desert-dwelling animals in the south of the country and, possibly, more injuries caused by pedigreed mastiffs in more affluent sections of the urban areas.

We lacked information on the dynamics surrounding the injury in terms of whether the pet was part of the child's household or if the attack was an unprovoked or provoked random encounter, whether parental negligence played a part in the child's vulnerability to attack, and whether preventative measures could or could not have been taken whatever the attacker. We recommend that these features be added to the reporting of the attack event in the ED in order to establish better guidelines for prevention of future injuries.

## Prevention

This analysis has pinpointed dogs as the major animal attacker of children in Israel. The preventive strategy will need to focus on public education of appropriate behavior around animals and stringent enactment and enforcement of canine leash laws and breed-specific legislation. Although hornet and scorpion stings are less prevalent, special emphasis should be placed upon their prevention since they have the highest hospitalization rate.

## ACKNOWLEDGMENTS

We thank Esther Eshkol for her assistance in writing this paper.

## BIOSKETCHES

**Michal Hemmo-Lotem, MD**, is a pediatrician and a graduate of the School of Educational Leadership in Jerusalem. She has established Beterem-The National Center for Children's Health and Safety in Israel and is currently the director (CEO). Beterem dedicates its work to childhood injury prevention, safety, and research. Dr. Hemmo-Lotem is the chairman of the National Committee for Childhood Injury Prevention and Safety and member of the National Council for Child Health and Pediatrics, the National Council for Health Promotion, and is involved in the public debate on childhood injury and safety. She is a lecturer at the School of Public Health, Haifa University. E-mail: mhemmo@beterem.org

**Yoav Barnea, MD**, is a Qualified Plastic Surgeon in the Department of Plastic and Reconstructive Surgery, Tel Aviv Sourasky Medical Center, Sackler School of Medicine, Tel Aviv University, Tel Aviv, Israel. Currently specializing in breast reconstruction. E-mail: barneay@netvision.net.il

**Claudia Jinich-Aronowitz, RD, MPH** is the Director of the Out-Reach Department in Beterem-The National Center for Safety and Health in Israel. She is one of the founders of the organization and for the past 9 years, has been dedicated to the promotion of safety through the work with health and educational professionals at all levels, as with the work with municipalities through out the country. E-mail: claudiaja@beterem.org

**Liri Endy-Findling, MPH,** was educated at the School of Public Health at the Hebrew University and is the Director of the Department of Research, Development and Policy at Beterem-the National Center for Children's Health and Safety, Israel. Beterem is an organization focused on injury prevention and safety promotion in childhood and Liri Endy-Findling is one of the leading figures in the organization. She has a rich background as a researcher from her experience at the National Research Institute for Social Sciences and Beterem. She leads public policy, programs development, research management, promotion of legislation and standards in the field of safety. E-mail: liri@beterem.org.il

**David Leshem, MD**, is a Qualified Plastic Surgeon in the Department of Plastic and Reconstructive Surgery, Tel Aviv Sourasky Medical Center, Sackler School of Medicine, Tel Aviv University, Tel Aviv, Israel, Also Qualified as pediatric Plastic Surgeon, in Toronto Sick Children Hospital.

**Arik Zaretski MD**, is a Qualified Plastic Surgeon in the Department of Plastic and Reconstructive Surgery, Tel Aviv Sourasky Medical Center, Sackler School of Medicine, Tel Aviv University, Tel Aviv, Israel. Presently specializing in Microsurgery.

**Joav Merrick, MD, MMedSci, DMSc,** is professor of child health and human development affiliated with the Center for Multidisciplinary Research in Aging, Zusman Child Development Center, Division of Pediatrics and Community Health at the Ben Gurion University, Beer-Sheva, Israel; the medical director of the Division for Mental Retardation, Ministry of Social Affairs, Jerusalem; and the founder and director of the National Institute of Child Health and Human Development. Dr. Merrick has numerous publications in the field of child health and human development, rehabilitation, intellectual disability, disability, health, welfare, abuse, advocacy, quality of life, and prevention. He received the Peter Sabroe Child Award for outstanding work on behalf of Danish Children in 1985 and the International LEGO-Prize (“The Children’s Nobel Prize”) for an extraordinary contribution towards improvement in child welfare and well-being in 1987. E-mail: jmerrick@internet-zahav.net. Website: www.nichd-israel.com

**Raphael Shafir MD**, is a retired chairman of the Department of Plastic and Reconstructive Surgery, Tel Aviv Sourasky Medical Center, Sackler School of Medicine, Tel Aviv University, Tel Aviv, Israel. Professor Shafir has dedicated his time to injury prevention and education, as well as cancer prevention. E-mail: shafir@post.tau.ac.il
